# Using Improved Principal Component Analysis to Explore Construction Accident Situations from the Multi-Dimensional Perspective: A Chinese Study

**DOI:** 10.3390/ijerph16183476

**Published:** 2019-09-18

**Authors:** Bo Shao, Zhigen Hu, Dawei Liu

**Affiliations:** State Key Laboratory of Water Resources and Hydropower Engineering Science, Wuhan University, Wuhan 430072, China; shaobo910@whu.edu.cn (B.S.);

**Keywords:** construction industry, accident situation, principal component analysis, multi-dimensional perspective

## Abstract

The improvement of the macro-level accident situation in the Chinese construction industry is currently an urgent task for the government due to the high accident rate. This study intends to use improved principal component analysis to explore the accident situations in the Chinese construction industry from a multi-dimensional perspective, aiming at providing targeted direction on the improvement of the accident situation for the government. Six composite indicators that can quantify the accident situation are firstly selected based on a wide review of the literature and interviews with safety experts, with the original data collected from China institutions. The classical principal component analysis is then improved to examine the correlations between indicators, and further to evaluate accident situations in China provinces. Finally, the features of accident situations are explored and analyzed from a multi-dimensional perspective. The findings show that the improved principal component analysis can retain more dispersion degree information of the original data. Meanwhile, three principal components including the accident frequency, trend, and severity were extracted to quantify the accident situation, and a hierarchical indicator system for the comprehensive evaluation of the accident situation was constructed to deeper understand multi-dimensional characteristics of China’s accident situations. Furthermore, there exist great regional differences of accident situations in Chinese provinces. From the overall perspective, the accident situation shows a declining trend from the western backward region to the highly developed eastern coastal region. This study provides a multi-dimensional perspective for the government to formulate safety regulations and improve the accident situation.

## 1. Introduction

The construction industry is globally notorious for poor accident records, especially in developing countries [1,2,3]. In China, the construction accident rate is closely related to safety management level of construction enterprises and safety regulatory system of the government [4,5]. Specifically, macro-safety regulations affect safety actions of the enterprises, and further affect the level of the accident occurrence in construction activities [3,6]. In recent years, a series of construction safety regulations have been launched and implemented from the national and provincial level [7,8,9,10,11]. Continuous efforts from the government have improved the accident situation (AS) in China’s construction industry [12]. However, the improvement effects vary slightly from region to region due to their different conditions such as construction safety regulation and the economic volume. Most notably, the entire industry experienced daily over 2.3 fatalities in 2018 [13]. Unbalanced and/or poor AS has brought great pressure to safety supervision of the government.

Currently, academia tends to explore the occurrence and evolution of construction accidents from the perspective of the accident itself [14,15,16]. This micro-level perspective can help to identify contributing factors of accidents by means of accident causation theories such as Swiss Cheese Model [17] and System-Theoretic Accident Model and Processes [18]. Although accident cause analysis is of significance for accident prevention [19,20], the government focuses more on exploring the AS of the entire industry, which can promote better understanding and improvement of the macro-level AS [21]. Under the circumstances, learning from better-performing regions and then applying successful experience into practice is an indispensable process of achieving maximum improvement of the AS [22]. Therefore, it is worthy for the government to clarify the ASs in China provinces from the macro-level perspective.

The AS is mostly employed to reflect the danger that accompanies the building industry during a given period [23]. Traditionally, AS can be measured by a series of basic indicators such as the number of accidents [24] and casualties [12]. However, these indicators cannot sufficiently reflect relative danger level in different provinces when considering some non-accident indicators such as construction practitioners [12]. To address this issue, some researchers are increasingly interested in developing composite accident situation indicator (CASI) [23]. Generally, CASI is a mathematical aggregation of two or more basic indicators from different perspectives. Currently, the commonly used CASIs involve the fatality rate per 100,000 full-time equivalent workers [25,26], the fatality rate per 100,000,000 yuan of GDP [12,27], and the fatality rate per 100,000 construction practitioners [23,24,28]. Notably, these CASIs can be individually used to evaluate ASs in different provinces, but the results could vary. The different results cannot provide coincident decision-making information for the government, maybe a comprehensive result will do, which is rarely focused by current researchers. Therefore, implementation of a comprehensive evaluation of the AS by means of combining different CASIs can be considered.

The first step towards a comprehensive evaluation is to find out whether the selected indicators are interrelated to each other. If so, this could make evaluation results biased due to information overlap [29,30]. To solve the problem, this study attempts to introduce principal component analysis (PCA) to examine the interrelations between indicators and further enable more accurate evaluation. This idea is mainly based on the fact that PCA is not only used to eliminate information overlap between indicators [30,31], but also is applied in the comprehensive evaluation through ranking PCA scores in multi-dimensional analysis [30,32].

Nowadays, PCA has been commonly applied in the field of dimensional reduction of original dataset [29,33,34,35]. In general, the original dataset mainly contains key information in the two aspects [36]: on the one hand, it contains the information of dispersion degree among all indicators, which is reflected by the variance; on the other hand, it contains the information of the correlations between all indicators, which is reflected by correlation coefficient matrix [36]. When standardizing the original dataset with large differences in the measured scales, it is essential for PCA to avoid the loss of these key information. However, the standardization process in classical PCA eliminates dispersion degree information contained in the original dataset, because it makes the variances of all indicators equal (all are equal to 1) [36,37]. Currently, few studies focused on the retention problem of key information of the original dataset in classical PCA [36,38]. Although Shang and Wang [36] improved the classical method, they limited the application of their method to positive values. Therefore, this study intends to improve the classical PCA to expand the value of application.

This study attempts to use improved PCA to explore the ASs in China construction industry and aims to provide targeted insights of safety regulation formulation for the government. The novelty of this study lies in three aspects: (1) Proposing improved PCA that can make the standardized dataset retain more key information; (2) exploring the underlying structure for evaluation indicators that can reveal the connotation of the ASs from different perspectives; and (3) proposing a multi-dimensional perspective that can contribute to better understanding of the AS in China construction industry. The rest of this paper is organized as follows. In the next section, CASIs are selected and the corresponding data are collected from authoritative institutions, followed by developing improved PCA. The findings of this research are presented in Section 3 and then they are discussed in Section 4. Finally, the conclusions are given in Section 5.

## 2. Materials and Methods

According to the website of the Ministry of Housing and Urban–Rural Development (MHURD) of China, fatal accidents have a dramatic increase in the recent years [13]. This poor record has posed great safety regulatory challenge to the government. In this study, the AS in China construction industry was chosen as the research case.

### 2.1. Selection of CASIs

Following a wide review of the literature and interviews with safety experts, many CASIs can measure the AS. Generally, the fatality rate per one accident (FOA) can reflect the average severity of fatal accidents with regards to a given period/region/accident type [12]. Meanwhile, the fatality rate per 100,000 construction practitioners (FCP) is widely applied in many countries, with an average of 4.2 in developed countries [23,28,39,40,41]. Moreover, Hola [24] held that building construction activities are always characterized by the variable values of obtained economic indicators in one region. Considering the rapidly growing development of China real estate industry in recent years, the floor area is an important economic indicator in the building industry. Increasingly, the fatality rate per 1,000,000 m^2^ of floor area (FFA) is regarded as a representative CASI [42]. Similarly, the fatality rate per 100,000,000 yuan of GDP (FGDP) is commonly used to evaluate the AS [43], and its value mainly ranges from 0.02 to 0.06 in developed countries [27]. Additionally, the AS has the temporal characteristic [44], which can reflect the changes in the number of fatal accidents and/or fatalities during given periods [24]. Accordingly, the trend of fatal accidents (TFA) and the trend of fatalities (TF) can help to describe ASs [25,45].

Based on China national conditions, there exist some limitations for some CASIs in the availability and acceptable quality of original data. For instance, the fatality rate per 100,000 full-time equivalent workers (FFEW) is widely accepted in many countries [25,26], but it is not a practical indicator in China due to the lack of statistical data. Moreover, some CASIs may contain similar information of ASs. For example, the connotation of the major fatal accident rate (MFA) is the similar to that of FOA [12]. The CASIs like FFEW and MFA were not considered in this study due to the two aspects of reasons. Based on essential principles of the indicator selection including measurability, representative and comparability [46], six CASIs were ultimately selected, as shown in Table 1.

### 2.2. Data Collection

According to the computing formulas in Table 1, each CASI is a mathematical combination of two basic indicators in the building industry. Therefore, original data of these basic indicators can be collected to indirectly obtain CASIs. Accordingly, accident information including the number of fatalities and fatal accidents came from the MHURD [13]. Moreover, other data including the number of construction practitioners, the floor area and GDP of the building industry were obtained from the National Bureau of Statistics (NBS) of China [47].

Considering the availability and acceptable quality of original data, the data in 2015 were chosen to evaluate ASs in China building industry. Because TFA and TF involved the number of fatal accidents and fatalities in 2014 respectively, seven sets of original data were ultimately collected to calculate the CASIs in this study. Eventually, the original data covered 30 provinces in China except Tibet, Hong Kong, Macao, and Taiwan, whose data were missing.

### 2.3. Improved PCA

PCA can employ the rotation of the coordinate system to convert a large dataset of possibly interrelated indicators into a smaller set of linearly uncorrelated indicators [29,31]. To clarify whether selected CASIs were interrelated to each other, PCA was introduced to examine the correlations between indicators. Moreover, the standardization process in classical PCA contributes to the loss of dispersion degree information of the original dataset, thus this study intends to improve the classical PCA to address the issue. The detailed steps of classical PCA are as follows:

**Step 1.** Standardize the original dataset. PCA is commonly applied to a dataset consisting of n samples with m indicators, thus the original dataset can be expressed as a matrix $X_{n\times m}$. Due to the different measured scales of the selected indicators, the dataset need be standardized to assure good comparability between indicators. Using Equation (1), $X_{n\times m}$ can be transformed into a standardized matrix $Y_{n\times m}$ with zero mean and unit variance.

(1) $$y_{ij}=\left(x_{ij}-{\bar{x}}_{j}\right)/S_{x_{j}}$$

where i=1,2,⋯,n, j=1,2,⋯,m, $x_{ij}$ is the jth indicator value of the ith sample in the original matrix $X_{n\times m}$, $x_{j}$ is the jth indicators of $X_{n\times m}$, ${\bar{x}}_{j}$ and $S_{x_{j}}$ are the mean and standard deviation of $x_{j}$, respectively, $y_{ij}$ is the standardized value of $x_{ij}$.

**Step 2** Calculate correlation coefficient matrix. Correlation coefficient matrix Φ can be used to reflect the correlation information between indicators, and it can be calculated by Equation (2).

(2) $$\Phi ={\left(\rho_{y_{j},y_{k}}\right)}_{m\times m}=\frac{1}{n-1}Y^{T}Y$$

where $y_{j}$ and $y_{k}$ are the jth and kth column vector of $Y_{n\times m}$, respectively, $\rho_{y_{j},y_{k}}$ is the correlation coefficient between $y_{j}$ and $y_{k}$, namely, correlation coefficient between the jth and kth indicator.

**Step 3** Calculate eigenvalues and eigenvectors. The eigenvalues and eigenvectors of Φ can be obtained by using |Φ−λE|=0. All eigenvalues arranged in descending order are $\lambda_{1},\lambda_{2},\cdots ,\lambda_{j},\cdots ,\lambda_{m}$. Each eigenvalue has its corresponding eigenvector. According to $\Phi b_{j}=\lambda_{j}b_{j}$, $b_{j}$ (the unit eigenvector corresponding to $\lambda_{j}$) is calculated and $\sum_{i=1}^{m}b_{ij}{}^{2}=1$. Therefore, $B=(b_{1},b_{2},\cdots ,b_{m})$ is a unit orthogonal matrix consisting of all the unit eigenvectors.

**Step 4** Determine principal components. The number of principal components is generally determined according to the criterion of ‘eigenvalue greater than one’ or ‘cumulative percentage variance greater than 80%’. The former is along with the Scree plot, and the latter is constructed by using $\alpha_{j}=\lambda_{j}/\sum_{j=1}^{m}\lambda_{j}$ and $\beta_{p}=\sum_{k=1}^{p}\lambda_{k}/\sum_{j=1}^{m}\lambda_{j}$, where $\alpha_{j}$ is percentage variance of ith principal component, $\beta_{p}$ is cumulative percentage variance of p principal components and p≤m. When $\beta_{p}\geq 80\%$ firstly appears, herein p principal components are selected.

**Step 5** Identify indicators belonging to determined principal components. The factor loading of each indicator on each determined principal component, namely, correlation coefficient $\theta_{jk}$ between the jth indicator and kth principal component is calculated by $\theta_{jk}=b_{jk}\sqrt{\lambda_{k}}$, where $\lambda_{k}$ is the eigenvalue corresponding to kth principal component, $b_{jk}$ is jth value of $b_{k}$. Only the indicator with $\left|\theta_{jk}\right|\geq 0.5$ is considered and it indicates that the jth indicator belongs to kth principal component. 

**Step 6** Calculate component scores. Every principal component is weighted linear combination of all indicators, and principal component scores ($f_{1},f_{2},\cdots ,f_{p}$) can be obtained by F=YB. Besides, the percentages of variation explained by each principal component are used as weights, which is calculated by $w_{k}=\lambda_{k}/\sum_{k=1}^{p}\lambda_{k}$. The comprehensive component score CF is thus obtained according to Equation (3).

(3) $$CF=\sum_{k=1}^{p}w_{k}f_{k}$$

Equation (1) in classical PCA makes the variance of each indicator equal to 1, which reduces the influence of dispersion degree differences on principal components [36,37]. Thus, principal components extracted from the standardized dataset could not fully reflect original information [38,48]. Based on this fact, an improved standardization method is proposed, as shown in Equation (4).

(4) $$z_{ij}=\left(x_{ij}-{\bar{x}}_{j}\right)/\varepsilon_{x_{j}}$$

where $\varepsilon_{x_{j}}=\max(x_{j})-\min(x_{j})$ and $\varepsilon_{x_{j}}>0$, $z_{ij}$ is the standardized value of $x_{ij}$. 

The mean and standard deviation of jth indicator ($z_{j}$) in $Z_{n\times m}$ can be obtained using Equations (5) and (6), respectively. According to Equation (7), $Z_{n\times m}$ and $X_{n\times m}$ have the same correlation coefficient matrix, which indicates that the improved standardization method keeps correlation information of all indicators. Importantly, dispersion degree differences of all indicators are partly retained according to Equation (6), and the classical PCA is to some extent improved.

(5) $${\bar{z}}_{j}=\sum_{i=1}^{n}\frac{x_{ij}-{\bar{x}}_{j}}{\varepsilon_{x_{j}}}/n=\frac{\sum_{i=1}^{n}\left(x_{ij}-{\bar{x}}_{j}\right)}{n\varepsilon_{x_{j}}}=0$$

(6) $$S_{z_{j}}=\sqrt{\frac{1}{n-1}\sum_{i=1}^{n}{\left(z_{ij}-{\bar{z}}_{j}\right)}^{2}}=\sqrt{\frac{1}{n-1}\sum_{i=1}^{n}{\left(\frac{x_{ij}-{\bar{x}}_{j}}{\varepsilon_{x_{j}}}\right)}^{2}}=\frac{S_{x_{j}}}{\varepsilon_{x_{j}}}$$

(7) $$\begin{array}{l} \rho_{z_{j},z_{k}}=\frac{C_{z_{j},z_{k}}}{S_{z_{j}}S_{z_{k}}}=\frac{1}{n-1}\sum_{i=1}^{n}\left(z_{ij}-{\bar{z}}_{j}\right)\left(z_{ik}-{\bar{z}}_{k}\right)/\left(S_{z_{j}}S_{z_{k}}\right)=\frac{1}{n-1}\sum_{i=1}^{n}\frac{\left(x_{ij}-{\bar{x}}_{j}\right)}{\varepsilon_{x_{j}}}\frac{\left(x_{ik}-{\bar{x}}_{k}\right)}{\varepsilon_{x_{k}}}/\left(\frac{S_{x_{j}}}{\varepsilon_{x_{j}}}\frac{S_{x_{k}}}{\varepsilon_{x_{k}}}\right)\\ =\frac{1}{n-1}\sum_{i=1}^{n}\left(x_{ij}-{\bar{x}}_{j}\right)\left(x_{ik}-{\bar{x}}_{k}\right)/\left(S_{x_{j}}S_{x_{k}}\right)=\rho_{x_{j},x_{k}} \end{array}$$

## 3. Results

### 3.1. Determination of Principal Components

The CASIs have different measurement scales, thus it is necessary to standardize their original dataset for enough comparability. The standardized dataset was obtained by Equation (4), then the correlation coefficient matrix was calculated by Equation (2), as shown in Table 2. According to Equation (7), correlation information of all CASIs was retained. Table 2 presented that FCP, FFA, and FGDP were significantly interrelated to each other, because correlation coefficients between them were more than 0.75 [49]. Similarly, there was a significant correlation between TFA and TF. Therefore, the correlations of some CASIs resulted in information overlap.

**Figure 1:**
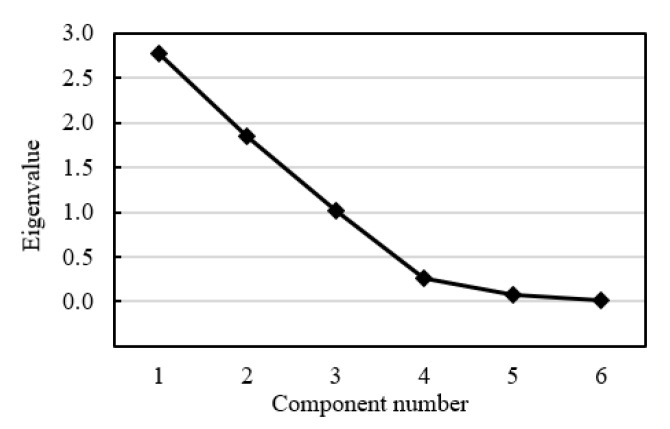
Scree plot of the eigenvalues of principal components.

To ensure the reasonable evaluation of the AS, it was essential to determine the principal components through eliminating insignificant information between CASIs. Based on Step 3 ~ Step 5 in Section 2.3, the eigenvalue, variance contribution and cumulative variance contribution corresponding to each component were obtained, respectively, as shown in Table 3. Herein, ‘Total’, ‘% of Variance’, and ‘Cumulative %’ represented the eigenvalue, percentage variance, and cumulative percentage variance, respectively. Table 3 showed that percentage variances of the first three principal components were 46.348%, 30.794%, and 16.972%, respectively, with a cumulative percentage variance of 94.114%. According to the criterion of ‘cumulative percentage variance greater than 80%’, three principal components should be selected [37,49]. Besides, the Scree plot in Figure 1 had a sharp descent in the eigenvalues from component 3 to component 4, and a level-off stage followed the component 3 after which the eigenvalues were less than 1. Generally, the component sequence number of 3 was called an inflection point, representing the total number of principal components [35,37]. Thus, selecting three principal components was an optimal solution.

According to Step 5, factor loadings corresponding to the three principal components were obtained, as shown in Table 4. Based on the criterion that the factor loading with greater than 0.5 should be considered [35,49], the first principle component mainly contained FCP, FFA, and FGDP, along with the second principal component containing TFA and TF, and the third principal component containing FOA. According to Step 6, the weights of the three principal components were calculated to be 0.493, 0.327, and 0.180, respectively. The detailed information of principal components was summarized in Table 5.

### 3.2. Connotation of Principal Components

A principal component generally consists of one or more indicators that have relatively high factor loadings, and its realistic connotation can be synthesized by the meanings of the included indicators [49]. Through analyzing the meanings of all CASIs, as well as their computing formulas in Table 1, FCP, FFA, and FGDP can reflect the frequency information of the AS in each province [23,39], thus the first principle component was interpreted to represent the accident frequency. Similarly, TFA and TF can reflect the trend information of the AS [24,25,45], then the second principal component was labeled as the accident trend; the third principal component was labeled as the accident severity, because FOA can reflect the severity information of the AS [12,50]. Therefore, the comprehensive evaluation of the ASs in China provinces was combined with different perspectives including the accident frequency, trend and severity.

Previous studies demonstrated that the accident risk is prone to the combination of the accident frequency [51,52], the accident severity [12,50] and the accident trend [1,25]. As a result, the accident risk can be regarded as the comprehensive expression of the accident frequency, severity, and trend. Based on this idea, the evaluation of the ASs in China provinces can be deemed to estimate their accident risk levels in the construction industry. Correspondingly, the six CASIs can be considered as a set of accident risk indicators.

**Figure 2:**
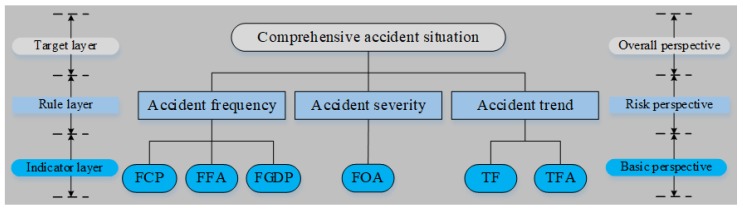
Hierarchical structure for accident situation (AS) evaluation.

Based on the core idea of PCA, the underlying structure of all CASIs is proposed for the first time. This structure for the AS evaluation involved the indicator layer, rule layer, and target layer [53], as shown in Figure 2. The factors on the indicator layer are called secondary indicators, and they can provide a basic perspective for the AS evaluation; the factors on the rule layer are called primary indicators, and they can provide a risk perspective; the factor on the target layer is called evaluation object, and it can provide an overall perspective.

From the basic perspective, the CASIs are commonly used separately for the evaluation of the AS, while their relative importance is rarely discussed. According to Table 3, the accident frequency accounted for 46.348% of the total variances, and it was relatively more important than the accident trend and severity. Similarly, the accident trend (30.794%) was relatively more important than the accident severity (16.972%). As presented in Table 5, the accident frequency, trend, and severity were weighted by 0.493, 0.327, and 0.180, respectively. Thus, the relative importance between CASIs was quantified from the risk perspective in this study, and the accident frequency has a greater impact on the evaluation of the AS.

### 3.3. AS in China Provinces

The component scores are standardized values on a linear scale [30,31,35], and they can provide a measurement of the position of a province in relation to other provinces for the ASs [35,54]. The greater the score is, the worse the AS is. According to the formula derivation in Section 2.3, the average score for each principal component is equal to 0, which represents the average level of the ASs in the provinces. Therefore, the provinces with positive scores have relatively worse AS compared to the average level; the provinces with negative scores have relatively better AS compared to the average level.

In this study, the component scores of principal components were obtained by using Step 6, as listed in the second to the fourth column of Table 6. Furthermore, the comprehensive scores can be calculated by Equation (8), depending on the weights of three principal components in Table 5. The comprehensive scores of all provinces were listed in the fifth column of Table 6.

(8) $$CF=0.493f_{1}+0.327f_{2}+0.180f_{3}$$

where $f_{1},f_{2},f_{3}$ were component scores of the three principal components, respectively.

On the whole, one-third of all provinces experienced relatively poor AS in the construction industry, as shown in Table 6. To deeper explore the ASs at a provincial level, the quantile classification method was selected to group the provinces into different grades based on comprehensive scores. This method usually places equal numbers of enumeration units into each grade [35,55]. Considering that 0 represented the average level, the provinces with positive scores and the provinces with negative scores were divided into two quantile categories respectively, as listed in the sixth column of Table 6.

In the four grades, Grade 1 represents the worst level of the AS and Grade 4 represents the best level in relative terms. From an overall perspective, five provinces including Qinghai, Ningxia, Hainan, Xinjiang, and Gansu had the worst ASs. Especially for Qinghai, its comprehensive score was 72.4% more than that in Ningxia, which ranked the second in all provinces. Conversely, the lower ranking provinces like Zhejiang and Beijing showed relatively good AS. The further results of the ASs in China provinces were obtained in the following subsection.

#### 3.3.1. AS from the Risk Perspective

**Figure 3:**
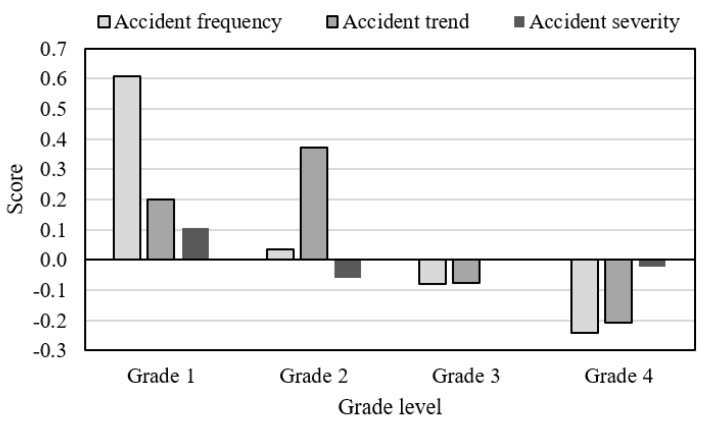
Distribution of the average scores for primary indicators in different grades.

From the risk perspective, the average scores were not evenly distributed in the four grades, as shown in Figure 3. It is clearly seen that Grade 1 provinces were generally accompanied by relatively high accident frequency and accident severity. Specifically, Qinghai belonged to Grade 1 due to the highest accident frequency, although its accident severity and trend were lower than the average level; Ningxia was classified into Grade 1, because it had higher scores for the three primary indicators in relative terms; Hainan, a Grade 1 province, had the second highest accident frequency and severity in all provinces; Xinjiang and Gansu squeeze into the tier of Grade 1 province, respectively depending on relatively high accident frequency and increasing trend.

Overall, Grade 2 provinces experienced relatively high accident trend. Especially for Shaanxi and Hebei, their percentage increase ranked the top two. Like Qinghai, Heilongjiang had relatively low accident severity and trend, but it suffered the fifth highest accident frequency. No matter from which one of the three risk perspectives, most of Grade 4 provinces had relatively good AS. Notably, Shandong and Sichuan saw the highest accident severity, although they belonged to Grade 4.

#### 3.3.2. AS from the Basic Perspective

**Figure 4:**
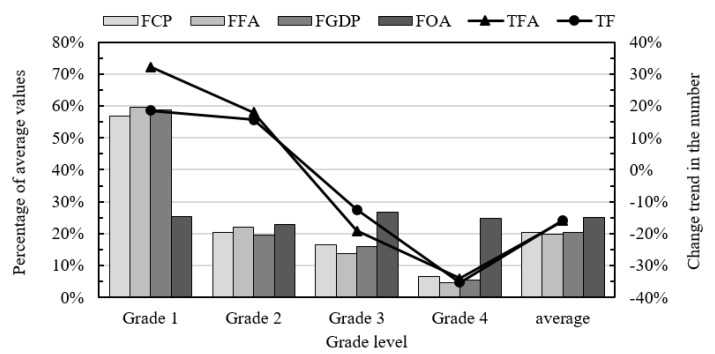
Distribution of secondary indicators in different grades.

To further explore the impact of CASIs on ASs in the provinces, the basic perspective was selected to analyze accident grade features, as shown in Figure 4. Considering different measurement scales of indicators, the left vertical axis represents the percentage of average values of indicators in the original dataset, involving FCP, FFA, FGDP, and FOA; the right vertical axis represents actual change trend in the number of fatal accidents and fatalities, involving TFA and TF.

Figure 4 shows that FCP, FFA, and FGDP have the similar change trend at the grade level. This indicates that the three indicators are largely related to each other, which is coincident with that presented in Table 2. From the view of FCP, an average value of 5.220 existed in Grade 1 provinces, and it was 2.8 times the average value of all provinces (1.885), as well as 8.8 times that in Grade 4 provinces (0.595). Specifically, FCPs in Grade 1 provinces including Hainan (6.729), Qinghai (6.434), Ningxia (5.556) and Xinjiang (5.255) ranked the top four, and they were far greater than those in the better-performing provinces such as Zhejiang (0.358), Shandong (0.433), Fujian (0.509) and Jiangsu (0.731). As for FGDP, the average values in Grade 2, Grade 3 and Grade 4 provinces were 0.008, 0.007, and 0.002 respectively, and all of them were less than that in all provinces (0.009). Notably, Grade 1 provinces performed badly due to an average FGDP of 0.025. Especially for Qinghai, its FGDP (0.064) was about 7 times the overall average value. With respect to FFA, Grade 1 provinces experienced the worst average value, which was approximately 2 times, 3 times, and 14 times more than that in Grade 2, Grade 3, and Grade 4 provinces respectively. Especially, the value in Qinghai (0.77) was more than the sum of that in Heilongjiang (0.29), Hainan (0.23), and Ningxia (0.21), which ranked the second, third, and fourth, respectively.

Besides, Figure 4 depicts that the changes of TFA and TF are approximately synchronous at the grade level, which is reflected as a significant correlation in Table 2. On the whole, the number of both fatal accidents and fatalities had a decreasing change of about 16%, thus the AS in 2015 was improved to some extent. However, the number of fatal accidents and fatalities in Grade 1 provinces increased by 32.3% and 18.6% respectively, with 17.9% and 15.7% for Grade 2 provinces respectively. Specifically, the percentage increases in Shaanxi (100%), Gansu (100%), Hebei (60%), and Xinjiang (55%) were more than 50% in the number of fatal accidents, and the percentage increases in Shaanxi (200%), Hebei (180%), and Gansu (100%) were no less than 100% in the number of fatalities. Noteworthy is the fact that Chongqing (a Grade 2 province) had the second highest number in both fatal accidents and fatalities, while it ranked the fifth in both TFA and TF (namely 38.7% and 30.3%), which were more than the average value in Grade 1 provinces.

With regards to FOA, it ranges from 1.12 to 1.31 in all grades. According to the classification of serious accidents [56], the range falls within the scope of Class 4 accident that involves no more than two fatalities, and Class 4 accidents are the predominant type of fatal accidents in all provinces. Relatively speaking, the provinces like Sichuan (2.25), Shandong (1.86), Guizhou (1.80), Ningxia (1.75), Hebei (1.75), Hainan (1.67), and Yunnan (1.62) had worse accident severity, with the FOA of more than 1.5. Especially for Sichuan, its FOA were between Class 4 accident and Class 3 accident.

#### 3.3.3. AS from the Regional Perspective

**Figure 5:**
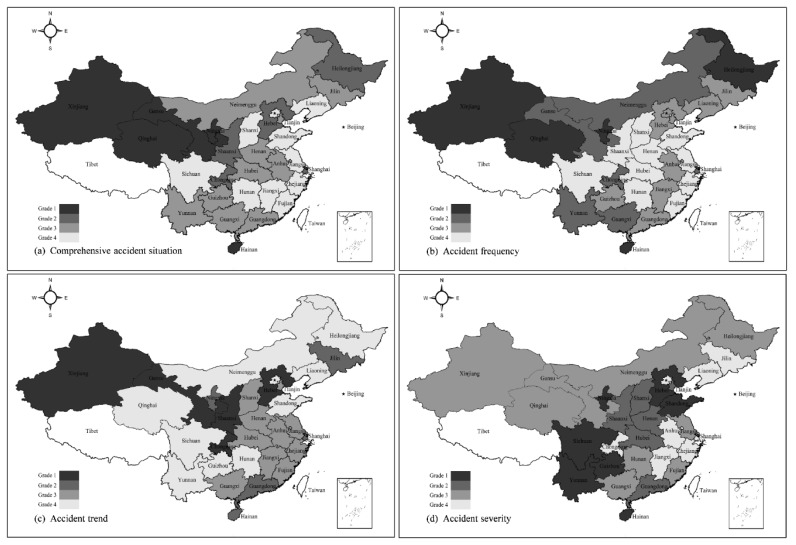
AS distribution in different regions. (**a**) AS from the overall perspective, (**b**) AS from the accident frequency, (**c**) AS from the accident trend, (**d**) AS from the accident severity. Note: Tibet, Hong Kong, Macao and Taiwan are not considered in this study.

Considering the differences of regional conditions in 30 provinces, the AS distribution are further considered to explore from a regional perspective. Based on the quantile classification method, the ASs from the risk perspective (the accident frequency, trend and severity) were classified into four grades respectively, as depicted in Figure 5.

From the overall perspective, Figure 5a shows that Grade 1 provinces including Qinghai, Ningxia, Xinjiang, and Gansu are largely concentrated in the northwestern region. Conversely, Grade 4 provinces such as Shandong, Jiangsu, Zhejiang, and Fujian are generally distributed in the eastern coastal area. From the view of the accident frequency, Figure 5b shows that the better-performing provinces are mainly concentrated in the central and eastern region, as well as the worse-performing provinces in the northern and southern region. From the view of the accident trend, Figure 5c shows that most of the southwest and northeast provinces had a relatively large decreasing trend in the number of fatal accidents and/or fatalities, while the relatively large increasing trend mainly existed in in the northwestern region. From the view of the accident severity, Figure 5d shows that the worse-performing provinces are mainly distributed in the southwestern region (e.g., Yunnan, Guizhou, and Sichuan) and Bohai Bay area (e.g., Shandong and Hebei). In addition, the municipalities including Beijing, Shanghai and Chongqing commonly performed better.

## 4. Discussion

### 4.1. Analysis of Improved PCA

**Figure 6:**
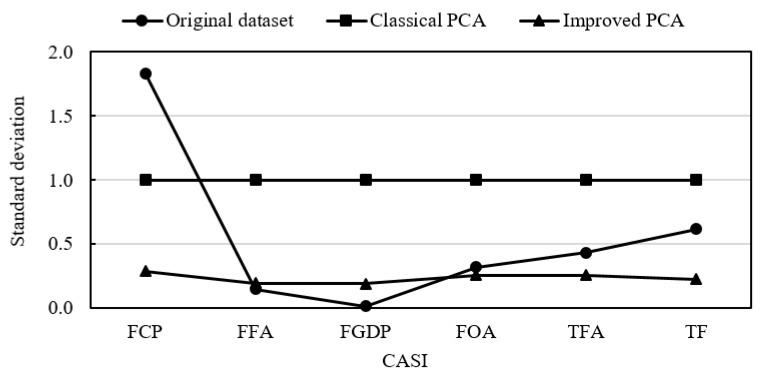
Standard deviations in three situations.

This study selects six CASIs that are commonly applied in China’s current conditions, and further uses improved PCA to evaluate the AS in China building industry. Considering the information overlaps that may exist in evaluation indicators, the improved PCA is used to examine the correlation between indicators in this study. The proposed method has two main advantages. On the one hand, it can make the standardized data retain more dispersion degree information of the original dataset compared to the classical PCA. Figure 6 shows the distribution of standard deviations of CASIs in three situations, namely the original dataset, classical PCA and improved PCA. The standard deviations of standard deviations of all the CASIs in three situations are 0.659, 0, and 0.039, respectively. Meanwhile, the correlation coefficient between standard deviations in the two situations of the original dataset and improved PCA is 0.785 (*p =* 0.032 < 0.05), with 95% confidence intervals. These indicate that the dispersion degree information of the original dataset was retained relatively much by using the improved PCA. In other words, the improved PCA can make evaluation results sounder.

On the other hand, the improved PCA has a wider range of applicability. It is well known that the standardization process in classical PCA eliminates dispersion degree differences of the original dataset due to its making the variance of each indicator equal to 1. To retain original information as much as possible, some studies are carried out to improve the standardization method in classical PCA [36,38]. For instance, Shang and Wang [36] used the formula $z_{ij}=x_{ij}/{\bar{x}}_{j}$ to standardize the original dataset. However, this method cannot retain the correlation information when the original dataset has both negative and positive values. With regards to the improved PCA in this study, it is well applicable for the standardization of TFA and TF that have both negative and positive values.

In addition, the improved PCA is well applied in the comprehensive evaluation of the AS through ranking the component scores. The rankings of ASs in the provinces in the classical PCA are slightly adjusted to be more reasonable, as listed in the seventh and eighth columns of Table 6. These findings reveal that the improved PCA is a feasible and sound method in multi-dimensional analysis.

### 4.2. AS Analysis from the Multi-Dimensional Perspective

Based on the improved PCA, this study mines a hierarchical indicator system for the comprehensive evaluation of the AS. This system provides a multi-dimensional perspective on the AS, involving an overall perspective, three risk perspectives and six basic perspectives. Compared to previous studies that focused more on a single aspect of the AS, this study can provide new insights for the government and those who care about the AS to better understand the AS.

From the overall perspective, the AS at a provincial level reveals a declining trend from the western region to the eastern coastal region, as shown in Figure 5a. The fact could be highly related to the socio-economic development level such as safety supervision input, medical services, educations, because the level in the western region generally has lagged far behind that in the eastern region [53]. The underdeveloped provinces could encounter relatively poor AS in China construction industry, while the developed provinces could be mostly better-performing regions. The findings are supported by the previous research [12]. Therefore, the western backward provinces should be paid more attention from the central and provincial government. Especially for the Grade 1 provinces including Qinghai, Ningxia, Xinjiang, and Gansu, they should learn from eastern coastal provinces and then apply successful experience into practice for achieving maximum improvement of the AS.

Notably, three risk indicators including the accident frequency, trend and severity provide a risk framework for better understanding of the AS. The indicators are weighted by 0.493, 0.327, and 0.180, respectively, and the relative importance between the indicators is quantified for the first time in this study. This finding indicates that the accident frequency plays a dominant role in the overall evaluation of the AS, and one province that has relatively high accident frequency could be prone to encounter poor AS on the whole. For instance, Qinghai and Heilongjiang belonged to worse-performing provinces due to their high accident frequency, although their accident trend and severity were below the overall average. Therefore, the government should pay more attention on the impact of the volume of construction industry (e.g., construction practitioners, GDP and the floor area) on the fatality rate in a province.

Specifically, FCP in China (1.9) is a relatively small value compared to 21 in Sub-Saharan Africa, 19.2 in South Africa, 9.7 in the US, 8.7 in Canada and 6 in the EU countries, respectively [28,39,40]. This reveals that FCP is at a relatively low level in China building industry. However, FCP in worse-performing provinces, especially Hainan, Qinghai, Ningxia and Xinjiang, is more than that in developed countries (4.2) [40]. This suggests that these provinces should learn more from better-performing regions to improve the poor situation, such as strengthening the education and training for practitioners.

FGDP varies greatly from province to province, and it ranges from 0.002 to 0.064. This implies that some provinces could pursue the growth of GDP at the expense of casualties. Poor accident record has hindered the sustainable development of the construction industry. Therefore, the provinces such as Qinghai, Hainan, and Heilongjiang should treat GDP rationally and formulate some reward and punishment policies based on the level of FGDP, as well as increasing safety input of the construction industry.

Currently, real estate companies frantically develop a great amount of building construction projects to meet the increasing needs of the rapid urbanization. However, they blindly pursue profits and attach few importance to construction safety management [57]. Considering that these companies have business in many provinces, they should be jointly supervised by multiple provinces and/or the central government, aiming to reduce FFA.

Although the overall accident trend has declined, some provinces like Gansu, Hebei, and Shaanxi may have out-of control in the increasing number of fatalities and fatal accidents. The government should firstly figure out the root reasons. As one and only municipality in Southwest China, Chongqing should attach great importance to the worse TFA and TF despite obvious advantages in economic development. With regards to the accident severity, provinces like Sichuan and Shandong pay more attention high-risk construction activities due to their high FOA, such as strengthening expert demonstration of the construction scheme and improving emergency rescue level.

This study shows the multi-dimensional feature of the ASs in China provinces. Sometimes, the worse-performing province from a certain perspective may perform better from other perspectives. Therefore, the multi-dimensional perspective provides a deeper understanding of the ASs for the government, as well as more reliable decision-making information for formulating macro-safety regulation in China construction industry. Moreover, Liu and Wu [27] concluded that the fatality rate is influenced by many factors such as GDP, the education level, the completeness of safety regulation and medical and health level, especially pointing out that the essential cause of high fatality rate is the disharmony of socio-technical system. Therefore, the worse-performing provinces should combine social systems and technical systems and further explore their interactions and relations under the condition of safety supervision [58].

### 4.3. Theoretical and Practical Implications

Several theoretical and practical contributions exist in this study. First, it contributes to the enrichment of the existing knowledge by proposing a hierarchical indicator system for the comprehensive evaluation of the AS in the construction industry. Second, it improves the applicability of the classical PCA by retaining key information of the original dataset, and can give a more accurate representation of evaluation results. Third, compared to traditional single perspective, it provides a multi-dimensional perspective for academic and practitioners to deeper understand the ASs in China construction industry. Fourth, it discusses the differences and features of the ASs in China provinces and provides some targeted insights of safety regulation formulation for the government. Fifth, the fatality rate is highly related to the socio-technical system in a region, and it suggests that exploring the interactions and relations between social systems and technical systems is an indispensable process of improving the macro-level AS. Sixth, it quantifies the relative importance between indicators measuring the AS for the first time and discovers the accident frequency as a dominant indicator. Finally, although this study was conducted in China, the proposed methods can be replicated in other regions or countries based on their actual conditions.

### 4.4. Limitations and Future Research

In this study, two main limitations need to be emphasized. One limitation is pertinent to the CASI. Although six CASIs contribute to sufficiently evaluate ASs, some other CASIs such as FFEW and MFA could be used to improve/extend the constructed hierarchical structure as long as they meet the requirements of essential principles of indicator selection, as well as the availability and acceptability of original data. The other involves research data. The original dataset in this study mainly comes from accident information issued in 2015. Using the one-year data to evaluate ASs only reflects the static characteristics of regional distribution, but cannot fully reflect the AS as a dynamic phenomenon. Thus, future works can be performed to collect the original data of two or more years for exploring dynamic properties of ASs during the particular period.

## 5. Conclusions

The high accident rate will probably continue to be one of the most significant safety challenges in the construction industry in the future. Governmental safety regulations play an important role in improving the macro-level ASs in China building industry. The study used improved PCA to evaluate the ASs in China provinces from a multi-dimensional perspective, aiming to assist the government to better understand the AS and further formulate safety regulations. As a tool for eliminating information overlaps, the improved PCA in this study cannot only retain more dispersion degree information in the original dataset, but also has a wider range of applicability. Meanwhile, the AS can be characterized by the accident risk that contributes to the construction of the hierarchical structure for the AS evaluation. The structure is treated as an extension of traditional AS evaluation framework, and captures a large number of accident information in a comprehensive way from multi-dimensional perspective including three risk perspectives and six basic perspectives. Based on China national conditions, the multi-dimensional perspective gives a deeper insight for comparative analyses of the ASs in China provinces. According to the evaluation results, the government can formulate pertinent macro-safety regulations to improve the macro-level AS. Noteworthy, the AS in one province could vary with different perspectives when using the multi-dimensional perspective, thus the government should treat the AS dialectically before learning from better-performing regions and then applying successful experience into practice.

## Figures and Tables

**Table 1:** Descriptions and computing formulas of selected composite accident situation indicators (CASIs).

CASI	Abbr.	Description	Computing Formula
Fatality rate per 100,000 construction practitioners	FCP	Reflecting the average fatality rate of construction practitioners.	$\frac{\mathrm{numberoffatalities}}{\mathrm{totalnumberofconstructionpractitioners}}\times 100,000$ ^(a)^
Fatality rate per 1,000,000 m^2^ of floor area	FFA	Reflecting the average fatality rate of accomplishing a certain floor area.	$\frac{\mathrm{numberoffatalities}}{\mathrm{grossfloorarea}}\times 1,000,000$ ^(b)^
Fatality rate per 100,000,000 yuan of GDP	FGDP	Reflecting the harmonious level between construction industry and economic development in one region.	$\frac{\mathrm{numberoffatalities}}{\mathrm{grossGDPofbuildingindustry}}\times 100,000,000$ ^(c)^
Fatality rate per one accident	FOA	Reflecting the average lethality of fatal accidents.	$\frac{\mathrm{numberoffatalities}}{\mathrm{numberoffatalaccidents}}$ ^(d)^
Trend of the number of fatal accidents	TFA	Reflecting changes in the number of fatal accidents during given periods.	$\frac{\mathrm{numberoffatalaccidents}\left(\mathrm{thisyear}-\mathrm{lastyear}\right)}{\mathrm{numberoffatalaccidents}\left(\mathrm{lastyear}\right)}$ ^(e)^
Trend of the number of fatalities	TF	Reflecting changes in the number of fatalities during given periods.	$\frac{\mathrm{numberoffatalities}\left(\mathrm{thisyear}-\mathrm{lastyear}\right)}{\mathrm{numberoffatalities}\left(\mathrm{lastyear}\right)}$ ^(f)^

*Notes*: (1) The computing formula ^(a)^ comes from the references such as Tupe [44] and Coates [23]. (2) For FFA, its connotation is similar to FGDP’s. Therefore, the computing formula ^(b)^ is proposed based on the same form. (3) The computing formulas ^(c)^ and ^(d)^ reference the literatures such as Shao, Hu, Liu, Chen, and He [12]. (4) The computing formulas ^(e)^ and ^(f)^ reference the literatures such as Dong, Fujimoto, Ringen, Stafford, Platner, Gittleman, and Wang [45]. (5) The measurement units of FCP, FFA, and FGDP are p/100,000 p (p = ‘person’), p/1,000,000 m^2^ and p/100,000,000 yuan, respectively. (6) For TFA and TF, the positive value represents an increase percentage; the negative value represents a decrease percentage; ‘0′ represents no changes. (7) For each CASI, the larger the value is, the relatively worse the AS is.

**Table 2:** Correlation coefficient matrix.

Pearson (Correlation)		CASI					
		FCP	FFA	FGDP	FOA	TFA	TF
**CASI**	**FCP**	1.000	0.788 **	0.800 **	−0.077	0.168	0.025
	**FFA**	0.788 **	1.000	0.992 **	−0.200	0.058	−0.063
	**FGDP**	0.800 **	0.992 **	1.000	−0.177	0.045	−0.064
	**FOA**	−0.077	−0.200	−0.177	1.000	−0.156	0.185
	**TFA**	0.168	0.058	0.045	−0.156	1.000	0.841 **
	**TF**	0.025	−0.063	−0.064	0.185	0.841 **	1.000

** Significance (*p* < 0.01).

**Table 3:** Total variance explained.

Component	Initial Eigenvalue			Extraction Sums of Squared Loadings		
	Total	% of Variance	Cumulative %	Total	% of Variance	Cumulative%
1	2.781	46.348	46.348	2.781	46.348	46.348
2	1.848	30.794	77.142	1.848	30.794	77.142
3	1.018	16.972	94.114	1.018	16.972	94.114
4	0.263	4.377	98.491			
5	0.083	1.386	99.878			
6	0.007	0.122	100.000			

**Table 4:** Eigenvectors and factor loadings of principal components.

CASI	Eigenvector			Factor Loading		
	P1	P2	P3	P1	P2	P3
FCP	0.536	0.035	0.167	0.893	0.048	0.168
FFA	0.583	−0.061	0.054	0.972	−0.083	0.055
FGDP	0.583	−0.066	0.081	0.973	−0.089	0.081
FOA	−0.156	0.052	0.951	−0.260	0.071	0.960
TFA	0.096	0.695	−0.201	0.160	0.944	−0.203
TF	−0.005	0.711	0.131	−0.008	0.966	0.132

**Table 5:** Detailed information of principal components.

Principal Component	Including CASIs	Connotation	Weight
P1	FCP, FFA, FGDP	Accident frequency	0.493
P2	TFA, TF	Accident trend	0.327
P3	FOA	Accident severity	0.180

**Table 6:** Principal component score for each province.

Province	P1 Score	P2 Score	P3 Score	Comprehensive Score	Classification	Ranking	
						Improved PCA	Classical PCA
Qinghai	1.454	−0.206	−0.029	0.643		1	1
Ningxia	0.468	0.221	0.390	0.373		2	2
Hainan	0.581	0.002	0.435	0.365	Grade 1	3	3
Xinjiang	0.420	0.297	−0.092	0.287		4	4
Gansu	0.111	0.687	−0.179	0.247		5	5
Shaanxi	−0.193	0.964	0.041	0.227		6	6
Hebei	−0.175	0.758	0.286	0.214		7	7
Heilongjiang	0.394	−0.238	−0.137	0.091	Grade 2	8	8
Chongqing	0.125	0.246	−0.287	0.090		9	9
Shanghai	0.032	0.136	−0.204	0.023		10	10
Guangdong	−0.126	0.163	0.002	−0.009		11	11
Yunnan	0.007	−0.205	0.258	−0.017		12	12
Guangxi	0.068	−0.069	−0.178	−0.021		13	13
Neimenggu	0.120	−0.206	−0.115	−0.029		14	14
Tianjin	−0.098	−0.054	0.024	−0.061	Grade 3	15	15
Hubei	−0.204	−0.002	0.066	−0.089		16	17
Jilin	−0.081	0.013	−0.295	−0.089		17	16
Guizhou	−0.150	−0.291	0.374	−0.102		18	18
Anhui	−0.072	−0.108	−0.200	−0.107		19	19
Henan	−0.249	−0.016	0.054	−0.118		20	20
Jiangxi	−0.172	−0.001	−0.249	−0.130		21	21
Fujian	−0.224	−0.111	−0.112	−0.167		22	22
Jiangsu	−0.193	−0.144	−0.179	−0.174		23	23
Shandong	−0.337	−0.251	0.387	−0.178		24	24
Sichuan	−0.408	−0.320	0.692	−0.181	Grade 4	25	25
Shanxi	−0.278	−0.205	0.081	−0.189		26	26
Hunan	−0.233	−0.222	−0.123	−0.210		27	27
Zhejiang	−0.239	−0.163	−0.243	−0.215		28	28
Liaoning	−0.190	−0.275	−0.202	−0.220		29	29
Beijing	−0.158	−0.401	−0.265	−0.256		30	30
